# Survival benefits of primary tumor surgery for synchronous brain metastases: A SEER‐based population study with propensity‐matched comparative analysis

**DOI:** 10.1002/cam4.5142

**Published:** 2022-08-14

**Authors:** Chengkai Zhang, Yuan Zhang, Deling Li, Wang Jia

**Affiliations:** ^1^ Department of Neurosurgery, Beijing Tiantan Hospital Capital Medical University Beijing China; ^2^ Beijing Neurosurgical Institute Beijing China

**Keywords:** brain metastases, primary tumor surgery, SEER, survival benefits

## Abstract

**Background:**

Evidence about the prognostic value of primary tumor surgery (PTS) in patients with brain metastatic malignancies is ambiguous and controversial. This study assessed the survival benefits of primary tumor surgery in patients with brain metastases (BMs).

**Methods:**

Adults patients with BMs that originated from lung, breast, kidney, skin, colon, and liver diagnosed between 2010 and 2018 were derived from the Surveillance, Epidemiology, and End Results database (SEER). Propensity score matching (PSM) was used to balance the bias between patients with or without PTS. Then the prognostic value of PTS was estimated by Kaplan–Meier analysis and Cox proportional hazard regression models.

**Results:**

A total of 32,760 patients with BMs secondary to non‐small cell lung cancer (NSCLC), small cell lung cancer (SCLC), breast cancer, renal cancer, melanoma, colorectal cancer, and liver cancer were identified from the database. After PSM at 1:1 ratio, PTS appeared to significantly prolong cause‐specific survival (CSS) time for patients with BMs secondary to NSCLC, breast cancer, renal cancer, and colorectal cancer (hazard ratio [HR] = 0.60 [0.53–0.68], 0.56 [0.43–0.73], 0.47 [0.37–0.60], and 0.59 [0.37–0.95], respectively, all *p* < 0.05). Patients with earlier T and N classifications, no extracranial metastasis, and cancer‐specific subtypes (adenocarcinoma in NSCLC, hormone receptor‐negative breast cancer) may derive more survival benefits from PTS when suffering from BMs.

**Conclusion:**

This population‐based study supported PTS could provide survival benefits for patients with BMs secondary to NSCLC, breast cancer, renal cancer, and colorectal cancer. More emphasis should be put on PTS of selected patients with BMs.

## Graphical abstract

This SEER‐based study assessed the survival benefits of primary tumor surgery (PTS) in patients with brain metastases (BMs). PTS appeared to significantly prolong cause‐specific survival (CSS) time for patients with BMs secondary to non‐small cell lung cancer, breast cancer, renal cancer, and colorectal cancer, rather than BMs from small cell lung cancer, melanoma, and liver cancer.

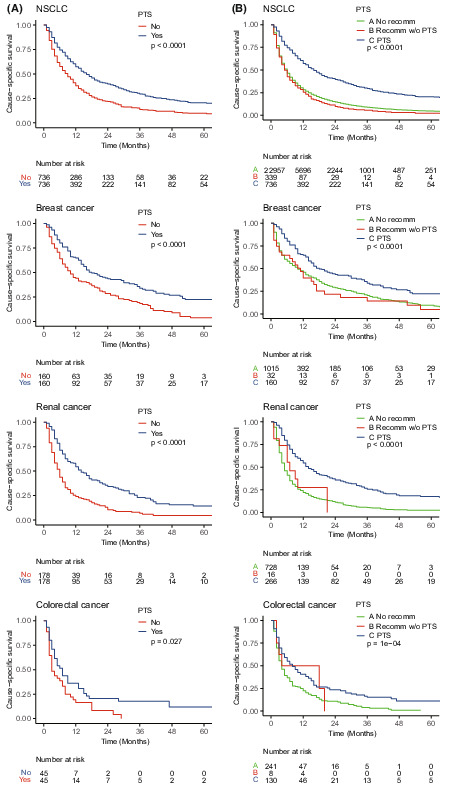

## 1 INTRODUCTION

Brain metastases occur in approximately 10%–40% of patients with cancer and are often associated with a dismal prognosis.^1^, ^2^ According to current national comprehensive cancer network (NCCN) guidelines, systemic therapy is the mainstream treatment for patients with metastatic (including brain metastatic) malignancies, while primary tumor surgery is still at a low recommendation level. For example, in advanced NSCLC with BMs, PTS is only considered for patients with early T&N classifications, good physical status, and well‐controlled intracranial lesions.^3^ In stage IV kidney cancer, cytoreductive nephrectomy for primary tumors can only be applied in selected patients.^4^ For BMs secondary to breast cancer, melanoma, and colorectal cancer, palliative salvage surgery at the primary site is only recommended for relieving symptoms or saving emergencies.^5^, ^6^, ^7^ PTS was not recommended for advanced small cell lung cancer (SCLC).^8^

Although incurable, advances in diagnosis and treatment have improved the survival of patients with BMs over the past decade^9^; and the appropriate local treatments of the primary tumors in BMs has become a major topic of debate. Several retrospective studies have shown the combined resection of primary tumors and brain metastases can improve survival time for selected patients with synchronous lung cancer and brain metastases.^10^, ^11^, ^12^, ^13^ However, these studies are limited to small samples, single center, single‐arm, single disease, and difficult to overcome selection bias. Furthermore, one population‐based study of the National Cancer Database (NCDB) has shown the survival of patients with BMs who received surgery of the primary site is longer than those who did not receive surgery. Nevertheless, it analyzed BMs of multiple origins together, regardless of the heterogeneity.^14^ Therefore, more information about the survival benefits of patients with BMs following PTS needs to be elucidated.

The Surveillance, Epidemiology, and End Results (SEER) database contains information on the BMs at diagnosis of primary malignancy and facilitates the investigation of BMs at a population‐based level.^2^ Accordingly, we used SEER data to provide a comprehensive assessment of PTS for patients with BMs.

## 2 METHOD

### 2.1 Study subject

The Surveillance, Epidemiology, and End Result Program (SEER) (18 registries, 2010–2018, November 2020 submission) was used for analysis,^15^ accessed through SEER*Stat (Version 8.3.9). SEER is a population‐based cancer registry developed by National Cancer Institute (NCI). It covers approximately 28.0% of the U.S. population and provides information about the demographic, disease, and treatment‐related covariates at diagnosis of primary malignancy.^16^ Primary cancer sites and histological types were identified according to the third edition of the International Classification of Diseases for Oncology (ICD‐O3). The TNM classification was coded according to the American Joint Committee on Cancer (AJCC) 7th edition.

Within the SEER incidence database, we identified synchronous brain metastases (patients were diagnosed with BMs at primary cancer) according to the following inclusion criteria: (i) BMs secondary to lung cancer, breast cancer, renal cancer, melanoma, colorectal cancer, and liver cancer, which constitutes the main types of BMs.^9^ (ii) the primary cancer was the first and only cancer diagnosis; (iii) ≥18 years old at the time of primary cancer diagnosis. The exclusion criteria were as follows: (i) diagnosed with cancer at autopsy or death certificate; (ii) the follow‐up was incomplete; (iii) TNM classification was unknown; (iv) patients were diagnosed as T0 classification; (v) whether the patient had brain, liver, bone, or lung metastases was unknown; (vi) whether the patient received PTS was unknown.

Eventually, we identified a population‐based sample of 32,760 BMs patients diagnosed between 2010 and 2018 for analysis, 73.3% secondary to NSCLC (*N* = 24,032), 16.3% secondary to SCLC (*N* = 5345), 3.7% secondary to breast cancer (*N* = 1207), 3.1% secondary to renal cancer (*N* = 1010), 1.9% secondary to melanoma (*N* = 634), 1.2% secondary to colorectal cancer (*N* = 379), and 0.5% secondary to liver cancer (*N* = 153). We performed the study according to the Declaration of Helsinki (2013 Revision). Since SEER is an open database providing anonymous information, no informed consent was needed.

### 2.2 Study design

We extracted variables as follows: demographic information (age at diagnosis, sex, race, time of diagnosis), clinical characteristics (primary site, TNM classification, differentiation grade, distant metastasis information), cancer‐specific variables (histology subtypes for lung cancer, renal cancer, and molecular subtypes for breast cancer), the first course of local therapy (primary tumor surgery, surgical recommendations, chemotherapy, and radiotherapy), and survival (overall survival time and cause of death). Cause‐specific survival (CSS) was defined as the time from diagnosis until death from specific primaries. Overall survival (OS) was defined as the time from primary tumor diagnosis until death.

Patients were categorized into those who had PTS (*N* = 1571) versus had not PTS (*N* = 31,189). 1:1 PSM was performed to reduce different degrees of bias. The prognosis for patients with PTS was assessed using survival analyses. To overcome the selection bias caused by surgeons who had a tendency toward patients with longer life expectancy, the recommendation of surgeons for PTS was also involved in analyses. Furthermore, survival benefits for surgical procedures were assessed. In order to demonstrate the synergy between surgery and chemoradiotherapy, 1:1 PSM about PTS was performed among patients who received chemoradiotherapy, and the effect of PTS was evaluated using survival analyses. Finally, the prognostic value of PTS for patients with different demographic and clinical characteristics was also analyzed in subgroups.

### 2.3 Statistical analysis

Categorical variables were compared by whether patients performed PTS using Pearson's chi‐squared test. Survival analyses were performed using the Kaplan–Meier method, and comparisons were made with the log‐rank method. Cox proportional hazard regression models were performed to obtain hazard ratios (HR) and 95% confidence interval (CI). Statistical analyses were performed using R software (version 4.1.0). A two‐side *p*‐value <0.05 was considered statistically significant. In addition, the proportion of PTS and other main treatments over the study period was estimated using JoinPoint analyses to allow for changes in trends.

## 3 RESULT

### 3.1 Baseline characteristics and PSM

In this cohort, 1571 patients underwent PTS, while 31,189 patients did not. Baseline characteristics for patients treated with or without PTS were shown in Table 1 and Table S1‐S6. In brain metastatic NSCLC, patients who received PTS tended to be of younger age, white race, earlier year of diagnosis, earlier T classification, no lymph node invasion, lower differentiation grade, less extracranial metastases (all *p* < 0.001); and they often simultaneously received chemotherapy and metastatic surgery (all *p* < 0.001) (Table 1). In other kinds of BMs, consistent tendencies were discovered in terms of age, year of diagnosis, T classification, N classification, and extracranial metastases. In addition, PTS was mainly performed in breast cancer with HR‐/HER2‐ subtype and advanced N classification, renal cancer patients with clear cell histology and advanced T classification, and colorectal cancer with advanced T and N classification. These findings suggested selection bias between PTS and non‐PTS groups, which may impact survival analysis.

**TABLE 1:** Baseline characteristics of non‐small cell lung cancer patients with brain metastases grouped by primary tumor surgery before and after propensity score matching

	Before matching			After matching		
	No PTS	PTS	*p* value	No PTS	PTS	*p* value
	*N* = 23,296	*N* = 736		*N* = 736	*N* = 736	
Age			<0.001			0.970
<60	7697 (33.0%)	310 (42.1%)		309 (42.0%)	310 (42.1%)	
60–69	8193 (35.2%)	282 (38.3%)		286 (38.9%)	282 (38.3%)	
>70	7406 (31.8%)	144 (19.6%)		141 (19.2%)	144 (19.6%)	
Sex			0.546			0.322
Female	10,989 (47.2%)	356 (48.4%)		376 (51.1%)	356 (48.4%)	
Male	12,307 (52.8%)	380 (51.6%)		360 (48.9%)	380 (51.6%)	
Race			<0.001			0.823
Black	3630 (15.6%)	74 (10.1%)		71 (9.65%)	74 (10.1%)	
White	17,275 (74.2%)	614 (83.4%)		622 (84.5%)	614 (83.4%)	
Other	2391 (10.3%)	48 (6.52%)		43 (5.84%)	48 (6.52%)	
Time of diagnosis			<0.001			0.917
2010–2012	7346 (31.5%)	282 (38.3%)		275 (37.4%)	282 (38.3%)	
2013–2015	7911 (34.0%)	239 (32.5%)		240 (32.6%)	239 (32.5%)	
2016–2018	8039 (34.5%)	215 (29.2%)		221 (30.0%)	215 (29.2%)	
Subtype			<0.001			0.664
Adenocarcinoma	15,327 (65.8%)	464 (63.0%)		477 (64.8%)	464 (63.0%)	
Bronchiolo‐alveolar	141 (0.61%)	39 (5.30%)		33 (4.48%)	39 (5.30%)	
Other NSCLC	4930 (21.2%)	138 (18.8%)		143 (19.4%)	138 (18.8%)	
Squamous carcinoma	2898 (12.4%)	95 (12.9%)		83 (11.3%)	95 (12.9%)	
T			<0.001			0.752
T1‐T2	8534 (36.6%)	415 (56.4%)		401 (54.5%)	415 (56.4%)	
T3‐T4	12,122 (52.0%)	289 (39.3%)		303 (41.2%)	289 (39.3%)	
Tx	2640 (11.3%)	32 (4.35%)		32 (4.35%)	32 (4.35%)	
N			<0.001			0.777
N0	4726 (20.3%)	321 (43.6%)		331 (45.0%)	321 (43.6%)	
N1‐N3	17,275 (74.2%)	398 (54.1%)		391 (53.1%)	398 (54.1%)	
Nx	1295 (5.56%)	17 (2.31%)		14 (1.90%)	17 (2.31%)	
Grade			<0.001			0.494
1	288 (1.24%)	17 (2.31%)		9 (1.22%)	17 (2.31%)	
2	1776 (7.62%)	174 (23.6%)		171 (23.2%)	174 (23.6%)	
3	5524 (23.7%)	324 (44.0%)		326 (44.3%)	324 (44.0%)	
4	250 (1.07%)	18 (2.45%)		14 (1.90%)	18 (2.45%)	
Unknown	15,458 (66.4%)	203 (27.6%)		216 (29.3%)	203 (27.6%)	
Lung metastasis			<0.001			0.199
No	17,328 (74.4%)	652 (88.6%)		668 (90.8%)	652 (88.6%)	
Yes	5968 (25.6%)	84 (11.4%)		68 (9.24%)	84 (11.4%)	
Liver metastasis			<0.001			1.000
No	18,932 (81.3%)	692 (94.0%)		692 (94.0%)	692 (94.0%)	
Yes	4364 (18.7%)	44 (5.98%)		44 (5.98%)	44 (5.98%)	
Bone metastasis			<0.001			0.883
No	14,767 (63.4%)	630 (85.6%)		627 (85.2%)	630 (85.6%)	
Yes	8529 (36.6%)	106 (14.4%)		109 (14.8%)	106 (14.4%)	
Chemotherapy			<0.001			0.957
No	10,768 (46.2%)	263 (35.7%)		261 (35.5%)	263 (35.7%)	
Yes	12,528 (53.8%)	473 (64.3%)		475 (64.5%)	473 (64.3%)	
Radiation			0.079			1.000
No	5576 (23.9%)	155 (21.1%)		154 (20.9%)	155 (21.1%)	
Yes	17,720 (76.1%)	581 (78.9%)		582 (79.1%)	581 (78.9%)	
Metastatic surgery			<0.001			1.000
No	19,993 (85.8%)	508 (69.0%)		507 (68.9%)	508 (69.0%)	
Yes	3303 (14.2%)	228 (31.0%)		229 (31.1%)	228 (31.0%)	

Abbreviations: NSCLC, non‐small cell lung cancer; PTS primary tumor surgery.

After 1:1 PSM, 2534 patients were included in the study, of which 1267 patients underwent primary tumor resection, and 1267 did not. As shown in Table 1 and Table S1‐S6, all baseline characteristics did not reach a significant difference between PTS performed or not (all *p* > 0.05).

### 3.2 Trends of PTS

**FIGURE 1:**
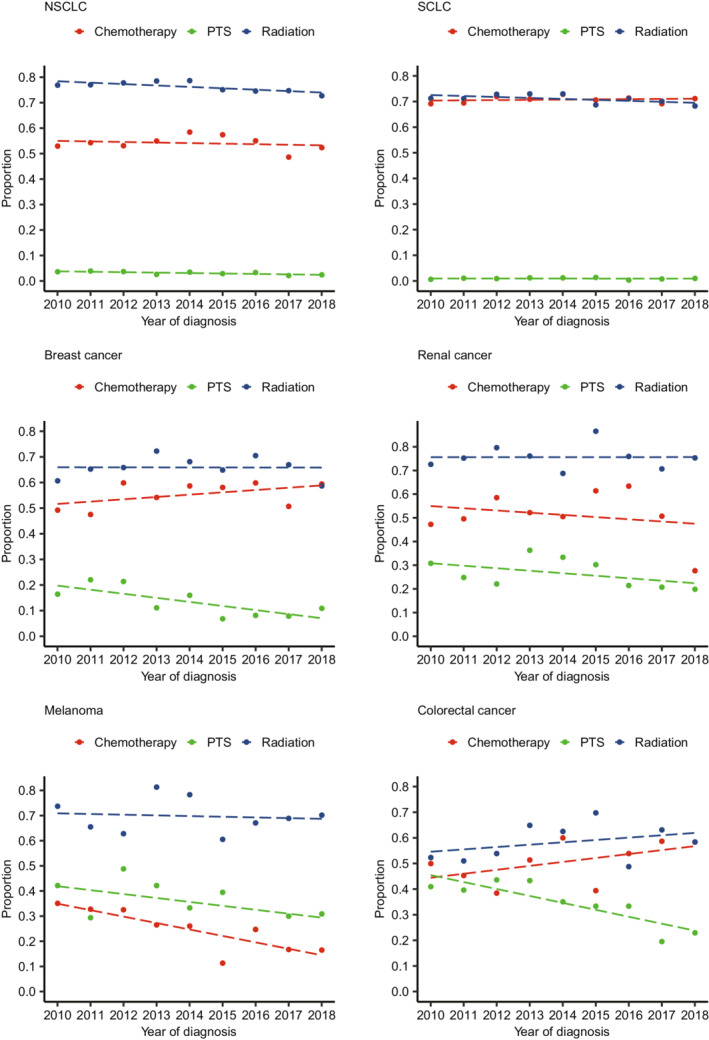
The treatment trends of patients with brain metastases from six primary tumor types between 2010 and 2018. NSCLC, non‐small cell lung cancer; PTS, primary tumor surgery; SCLC, small cell lung cancer.

From 2010 to 2018, the proportion rate of PTS for patients with BMs remained low and declining in six main primary tumor types (Figure 1). The PTS rates of different malignancies was 3.1% for NSCLC (736/24032), 0.9% for SCLC (49/5345), 13.3% for breast cancer (160/1207), 26.3% for renal cancer (266/1010), 34.4% for melanoma (218/634), 34.3% for colorectal cancer (130/379), and 7.8% for liver cancer (12/153). In addition, radiotherapy and chemotherapy remained the mainstem treatment for patients with BMs (Figure 1).

### 3.3 Survival estimates

**FIGURE 2:**
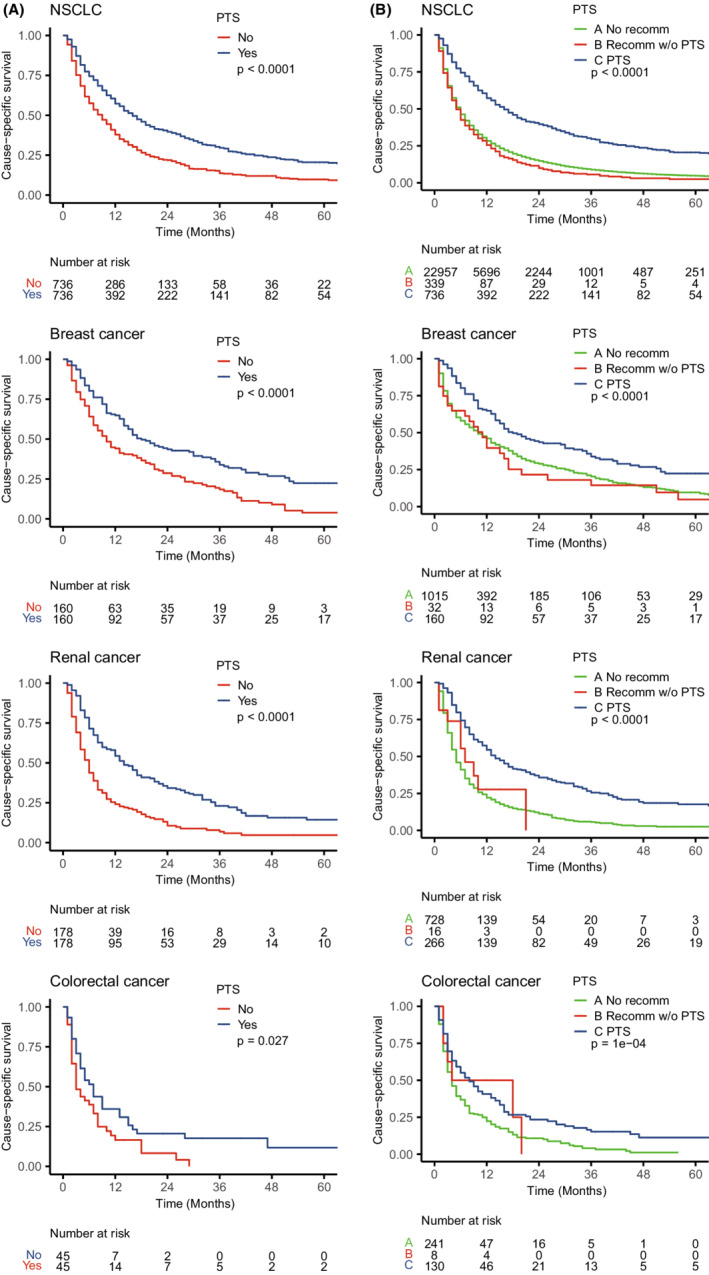
Kaplan–Meier curves of cause‐specific survival by primary tumor surgery after propensity score matching depending on the primary tumor types. Individual surgeon selection bias was not excluded (A) or excluded (B). The *p* values were calculated by log‐rank tests. NSCLC, non‐small cell lung cancer; PTS, primary tumor surgery; recomm, recommended; SCLC, small cell lung cancer; w/o, without.

The median follow‐up duration for the entire cohort was 5 months (interquartile range, 10 months). After PSM, PTS was associated with improved cause‐specific survival among patients with BMs secondary to NSCLC (HR: 0.60, 95% CI: 0.53–0.68, *p* < 0.001), breast cancer (HR: 0.56, 95% CI: 0.43–0.73, *p* < 0.001), renal cancer (HR: 0.47, 95% CI: 0.37–0.60, *p* < 0.001), colorectal cancer (HR: 0.59, 95% CI: 0.37–0.95, *p* = 0.029) (Figure 2). However, PTS did not provide survival benefits for those with BMs secondary to SCLC (HR: 0.65, 95% CI: 0.42–1.01, *p* = 0.058), melanoma (HR: 0.79, 95% CI: 0.55–1.12, *p* = 0.182), and liver cancer (HR: 0.80, 95% CI: 0.34–1.88, *p* = 0.606) (Figure S1). Consistently, PTS can prolong overall survival for patients with BMs secondary to NSCLC (HR: 0.61, 95% CI: 0.54–0.68, *p* < 0.001), breast cancer (HR: 0.58, 95% CI: 0.45–0.75, *p* < 0.001), renal cancer (HR: 0.46, 95% CI: 0.37–0.59, *p* < 0.001), colorectal cancer (HR: 0.59, 95%CI: 0.38–0.93, *p* = 0.023), rather than SCLC (HR:0.69, 95% CI: 0.44–1.07, *p* = 0.096), melanoma (HR: 0.80, 95% CI:0.57–1.13, *p* = 0.215), and liver cancer (HR: 0.80, 95%CI: 0.34–1.88, *p* = 0.606) (Figure S2). Patients who were recommended for PTS by surgeons but did not perform had similar cause‐specific survival to patients who were not recommended for PTS, and the survival was worse than those who underwent PTS (Figure 2, Figure S1). This finding further demonstrated that the survival benefits were caused by PTS rather than surgeons' selection bias.

**FIGURE 3:**
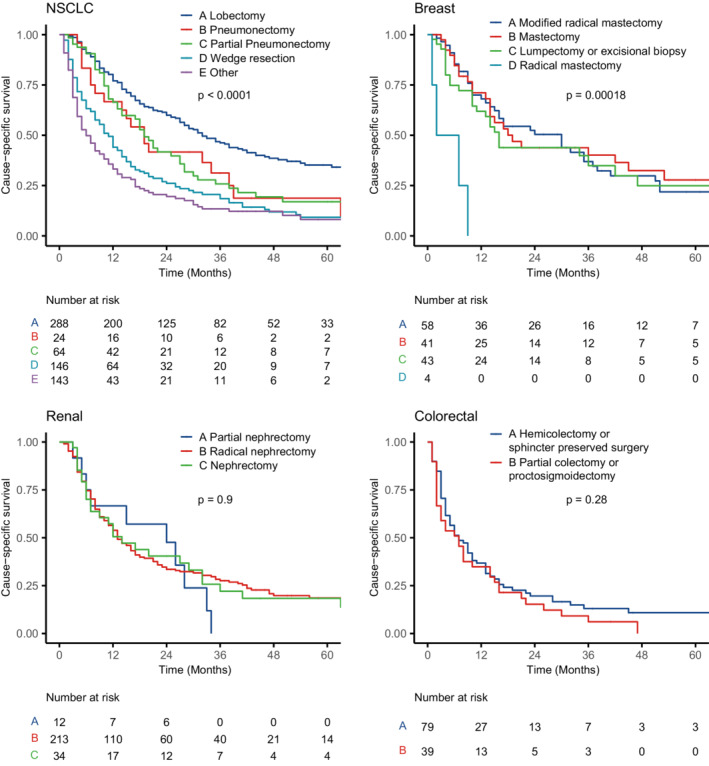
Kaplan–Meier curves of cause‐specific survival by primary tumor surgical procedures after propensity score matching depending on the primary tumor types. The *p* values were calculated by log‐rank tests. NSCLC, non‐small cell lung cancer.

In brain metastatic NSCLC, patients who had undergone lobectomy had longer cause‐specific survival, followed by pneumonectomy and partial pneumonectomy. Wedge resection and other surgical procedures were related to a relatively poor prognosis (Figure 3). In breast cancer patients with BMs, modified radical mastectomy and mastectomy can achieve a better prognosis. The same trend was notable, but to a lesser degree, for patients with lumpectomy or excisional biopsy. However, patients who received the radical mastectomy had a poor prognosis (Figure 3). The surgical procedures did not show different prognoses in brain metastatic renal cancer and colorectal cancer (Figure 3).

### 3.4 The role of PTS in BMs management strategies

**FIGURE 4:**
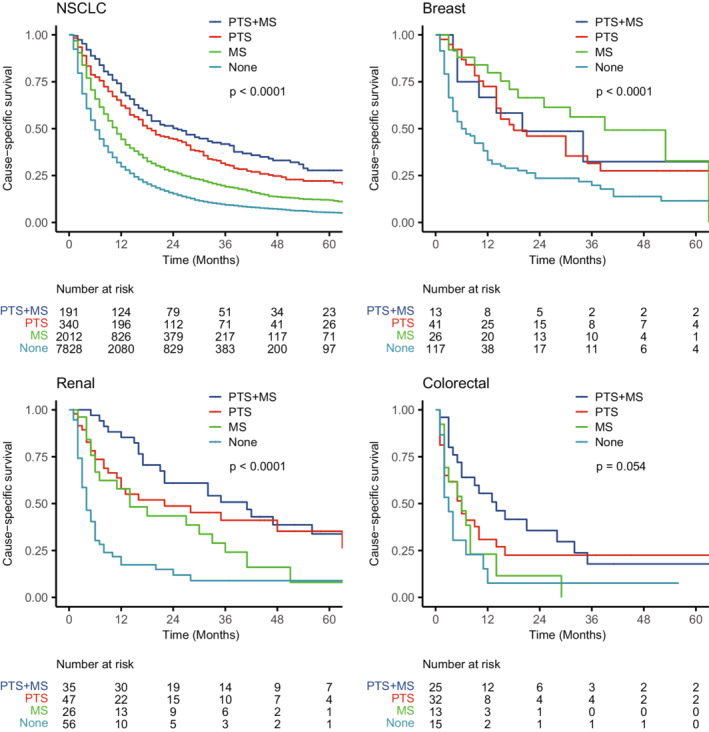
Kaplan–Meier curves for cause‐specific survival of patients only with metastases in brain by surgical sites depending on the primary tumor types. The *p* values were calculated by log‐rank tests. MS, metastatic surgery; NSCLC, non‐small cell lung cancer; PTS, primary tumor surgery.

Among patients only with metastases in brain, the combination of PTS and neurosurgical resection was associated with a superior prognosis in NSCLC and renal cancer (Figure 4). Among patients with metastases both in brain and other organs, the combination of PTS and metastatic surgery was also associated with a better prognosis in NSCLC and renal cancer (Figure S3).

**FIGURE 5:**
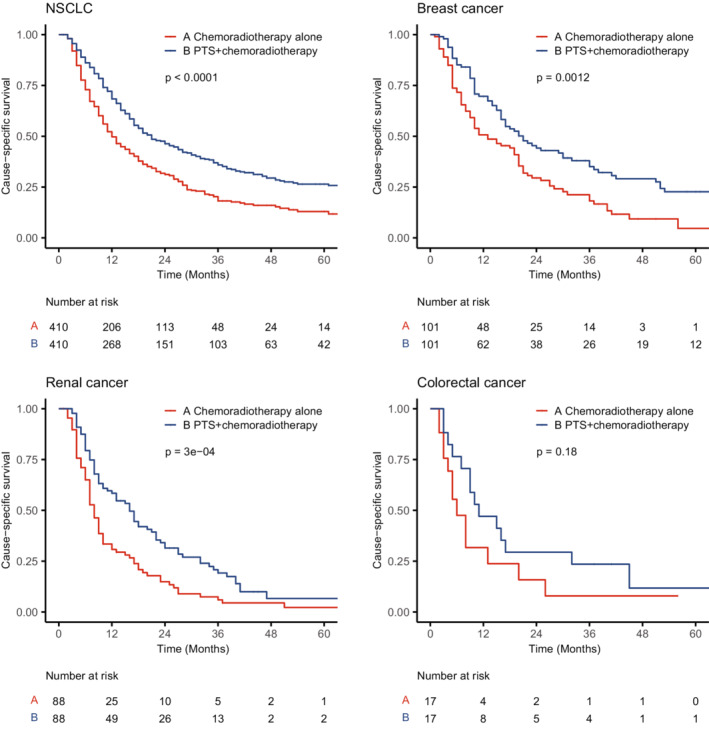
Kaplan–Meier curves of cause‐specific survival by treatment after propensity score matching depending on the primary tumor types. The *p* values were calculated by log‐rank tests. NSCLC, non‐small cell lung cancer; PTS, primary tumor surgery.

Compared with chemoradiotherapy alone, combined PTS and chemoradiotherapy can derive better prognosis in patients with BMs secondary to NSCLC, breast cancer, and renal cancer. There was also a consistent trend in BMs from colorectal cancer; however, limited to a small sample size after PSM, the difference was not statistically significant (log‐rank, *p* = 0.18) (Figure 5).

### 3.5 Subgroup analyses

**FIGURE 6:**
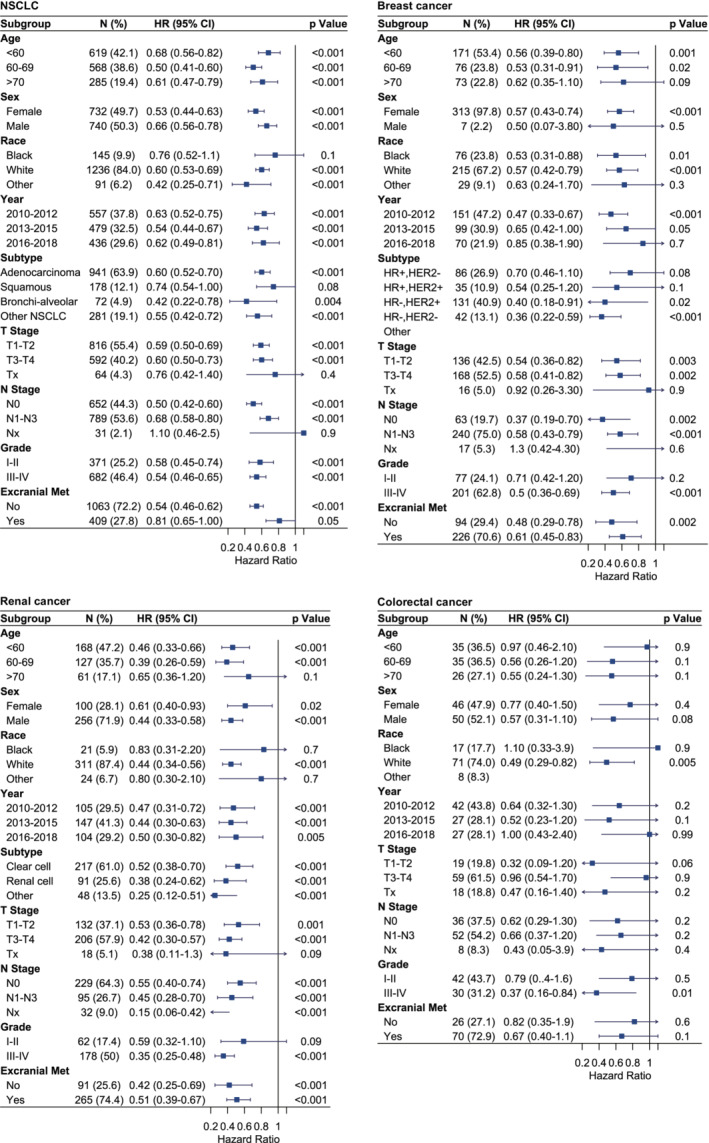
Subgroup analyses estimating the prognostic value of primary tumor surgery grouped by patients with different demographic and clinical characteristics after propensity score matching depending on the primary tumor types. Cox proportional hazard regression for cause‐specific survival was presented as the hazard ratio and 95% confidential interval. CI, confidential interval; HER2, human epidermal growth factor receptor 2; HR, hazard ratio; HR, hormone receptor; NSCLC, non‐small cell lung cancer.

Subgroup analyses evaluating the prognostic value of PTS for patients grouped by primary tumor sites, demographic and clinical characteristics after PSM were presented in Figure 6. Patients with N0 classification and no extracranial metastasis can generally benefit more from PTS. For BMs from NSCLC, subgroups of female, adenocarcinoma, and earlier T classification had greater survival benefits from PTS. For BMs from breast cancer, PTS can more obviously improve OS in patients with hormone‐receptor negative subtypes and earlier T classification. For BMs from renal cancer, T3‐T4 classification was a favorable predictor for PTS. For BMs from colorectal cancer, the small sample size limited the subgroup analysis, although PTS was associated with a favorable trend in most subgroups.

## 4 DISCUSSION

Primary tumor surgical interventions can provide immediate and effective relief from symptomatic mass effects, enable the removal of large tumors that require decompression, and help to confirm or establish a diagnosis, which is important for guiding subsequent targeted therapy. The value of PTS for metastatic solid cancer has been widely discussed. Previously studies suggested PTS can prolong survival time for screened metastatic cancer patients, including NSCLC,^17^, ^18^ breast cancer,^19^, ^20^, ^21^ kidney cancer,^22^ and colorectal cancer.^23^ The progress of treatment and the extension of survival time of BMs provided potential opportunities for aggressive local treatments,^9^, ^24^ which promoted us to reassess the value of PTS. In this population‐based study, despite the PTS was rarely performed, it can provide significant survival benefits for patients with BMs secondary to NSCLC, breast cancer, renal cancer, and colorectal cancer, rather than those who had SCLC, melanoma, and liver cancer, which showed that the PTS has not been fully utilized.

Our analyses were consistent with previous studies. The local control of primary tumors was an important prognostic factor in BMs.^25^ One population‐based study showed that the median survival time of patients with BMs who received PTS has prolonged than those who did not receive PTS (20 vs. 9 months).^14^ Several single‐institution cohorts demonstrated that thoracic surgery for NSCLC with only BMs was associated with longer survival time.^18^, ^26^ Another study showed that breast cancer patients in M1c (brain metastasis involved regardless of the number of metastatic sites or multiple metastatic lesions including liver) can still gain survival benefits from PTS (HR, 0.59; 95% CI, 0.44–0.80, *p* < 0.001).^27^ In a study of 278 breast cancer patients with only brain metastasis, the survival benefit of PTS counted for little, although PTS showed a favorable trend in OS (*p* = 0.0452) and CSS (*p* = 0.0489).^28^ We also noticed that studies about surgical treatment for patients with BMs were mainly focused on neurosurgical resection of limited numbers of intracranial lesions,^29^ while PTS for such patients lacked adequate analyses. Both previous and present studies indicated that PTS was as important as neurosurgical resection.

In our study, curative resection (lobectomy for NSCLC, mastectomy for breast cancer, and nephrectomy for renal cancer) was generally favored. However, more aggressive resection (pneumonectomy for NSCLC, radical mastectomy for breast cancer) was not associated with favorable prognosis. On the one hand, PTS for advanced solid tumors can avoid tumor‐related complications and prolong survival time^30^; on the other hand, PTS was also combined with the risk of perioperative mortality.^31^ Therefore, the benefits and risks of PTS should be fully considered before making a personalized decision.

In advanced NSCLC, renal cancer, and colorectal cancer, although total resection of all lesions in patients was usually difficult, the combination of PTS and neurosurgical resection can derive a better prognosis for patients only with metastases in brain. It was not surprising, since previous similar studies have shown surgical resection of both lung primary and brain metastatic tumors can prolong survival time, and the one‐year survival rate ranged from 35% to 80%.^10^, ^12^, ^26^, ^32^ In the subset of breast cancer, the combination of PTS and neurosurgical resection had a similar prognostic effect with PTS alone. At the same time, a NCDB‐based retrospective study also observed similar OS between combined lumpectomy/mastectomy and neurosurgical resection versus lumpectomy/mastectomy alone (median OS, 16.2 vs. 16.5 months).^33^ Considering a variety of factors (i.e., the mass effect of intracranial lesions, the extent of tumor resection) could affect OS, this finding remains to be further demonstrated.

Although PTS is an important treatment option for patients with BMs, the combination of approaches the brain metastases space will continue to provide an evolving landscape of treatment options. Previous studies discovered PTS could minimize the tumor burden and play a synergetic role with subsequent immunotherapy,^14^, ^18^ and targeted therapy^18^, ^34^ or chemotherapy.^27^, ^35^ We found that primary tumor surgery combined with chemoradiotherapy can get a better prognosis. Therefore, multidisciplinary approaches are strongly recommended for patients with BMs.^9^, ^36^, ^37^, ^38^

PTS was relatively more beneficial for patients who were diagnosed with BMs with earlier T or N classification, no extracranial metastasis, and cancer‐specific subtypes. A retrospective study of 86 cases also found adenocarcinoma histologic type, no extracranial metastasis are positive prognostic factors in NSCLC with BMs after PTS.^39^ In BMs from HR‐positive breast cancer, endocrine therapy had favorable efficacy^40^; and the present study showed the benefits of PTS were more obvious among HR‐negative breast cancer patients. Therefore, PTS may reflect a preferable management strategy for patients with particular clinical characteristics, given the generally improved overall prognosis that these patients display.

We would like to acknowledge that there were several limitations in this study. First, the time to metastasis on patients affects patient demographics and treatment strategies.^41^ In this study, we focused on the patients with synchronous brain metastases, that is, patients are diagnosed with a primary tumor and metastases simultaneously. As for metachronous metastatic, that is, developing metastases during follow‐up is a unique subset of brain metastases that require special consideration during clinical decision‐making. Secondly, since the SEER database only provides prognostic information on overall survival and cause‐specific survival, we were unable to analyze the local control rate, progression‐free survival, and quality of life, which were also important for the evaluation of treatment effect. Thirdly, the PSM analyses cannot balance unmeasured characteristics and confounders between groups. As a result, remaining unmeasured confounding variables may lead to biased results. For example, the SEER database does not provide information about gene mutation, detailed chemotherapeutic drugs, targeted therapy, and surgical procedures for metastases, which all have important implications for predicting individual patient prognosis. Lastly, in the survival analysis of multiple categorical variables, we cannot match confounding factors by paired PSM. Therefore, the risk of bias may increase.

## 5 CONCLUSION

Brain metastases have greatly improved over the past decade owing to advances in imaging, radiotherapy, targeted agents, immunotherapy, and genomics.^9^ In this population‐based analysis, PTS (especially curative surgery) was associated with better prognosis in NSCLC, breast cancer, renal cancer, and colorectal cancer patients with synchronous BMs, rather than SCLC melanoma, and liver cancer. The result of our study suggested that patients with earlier T and N classifications, no extracranial metastasis, and cancer‐specific subtype (adenocarcinoma or bronchi‐alveolar carcinoma in NSCLC, hormone receptor‐negative breast cancer) may derive more benefit from PTS, when suffered from BMs. Our study provided evidence for surgical decision‐making and clinical trial recruitment.

## AUTHOR CONTRIBUTIONS

Wang Jia and Deling Li designed the project. Chengkai Zhang and Yuan Zhang analyzed data and wrote the manuscript. All authors reviewed the manuscript.

## FUNDING INFORMATION

National Natural Science Foundation of China, Grant. 82071996.

## CONFLICT OF INTEREST

All authors declare no conflict of interest.

## ETHICAL APPROVAL STATEMENT

This was a retrospective study base on the Surveillance, Epidemiology, and End Results database (SEER), which provides anonymous information. Therefore, ethics approval or informed consent was not required.

## Supporting information


Appendix S1

## Data Availability

The data that support the findings of this study are available from the corresponding author upon reasonable request.
