# Supplementary material for: Survival benefits of primary tumor surgery for synchronous brain metastases: A SEER‐based population study with propensity‐matched comparative analysis
Source: Cancer Med. 2022 Aug 14;12(3):2677–90. doi: 10.1002/cam4.5142 (PMC9939173; doi:10.1002/cam4.5142)
Supplement: Supplementary file 1 — Appendix S1 [file CAM4-12-2677-s001.docx]

**Supplement material**


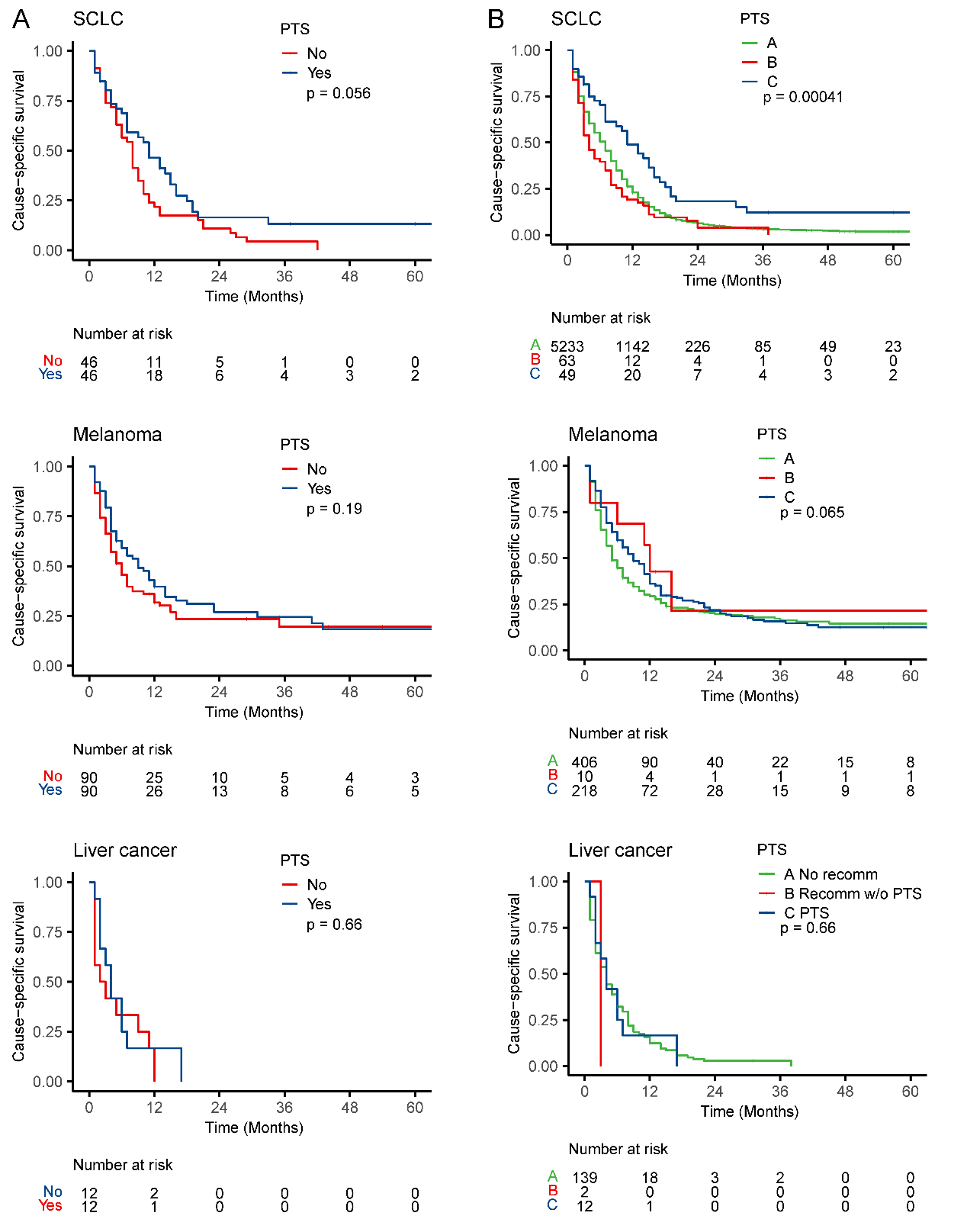


Figure S1: Kaplan-Meier curves of cause-specific survival by primary tumor surgery after propensity score matching depending on the primary tumor type. Individual surgeon selection bias was not excluded (A) or excluded (B). PTS primary tumor surgery, recomm recommended, w/o without.


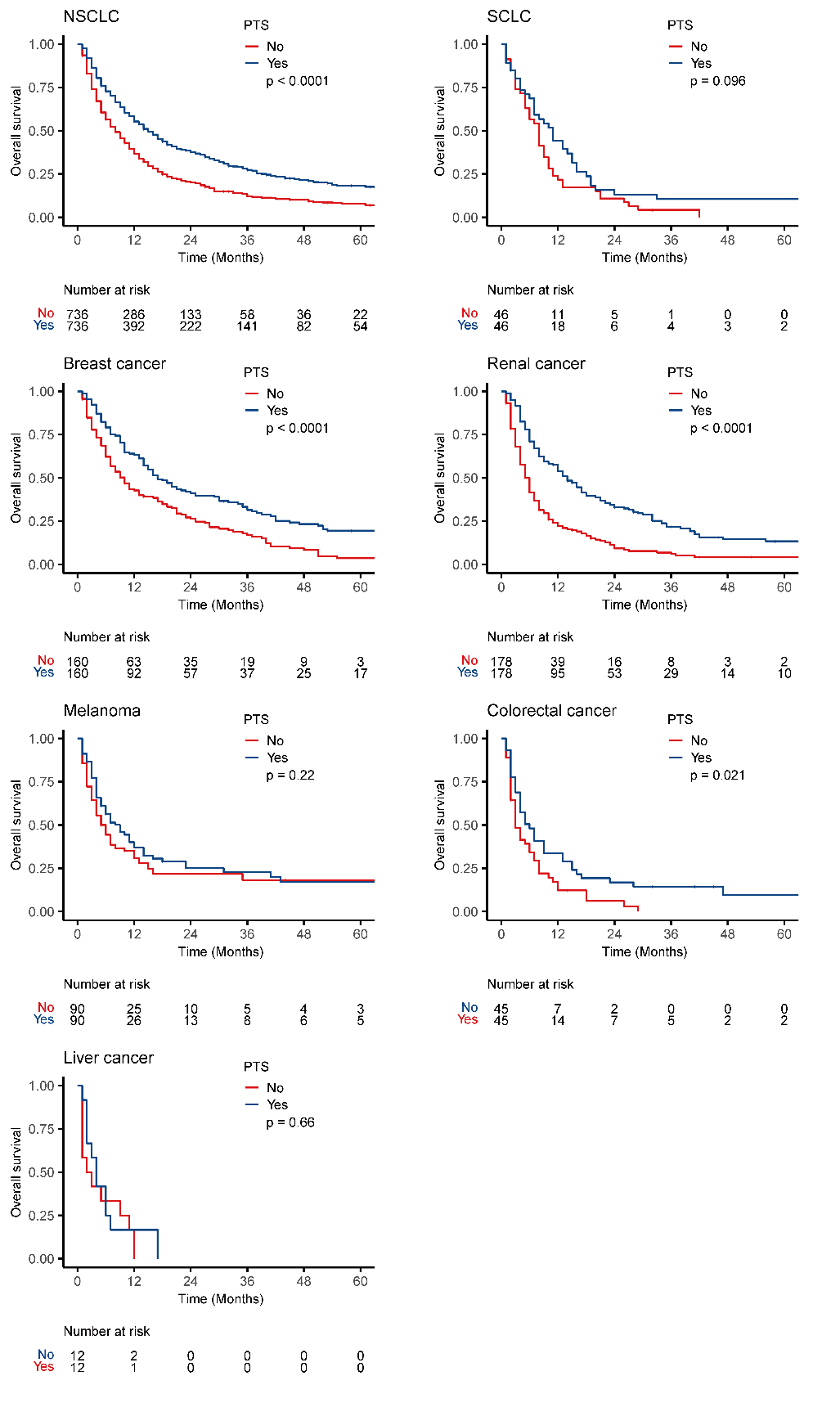


Figure S2: Kaplan-Meier curves of overall survival by primary tumor surgery after propensity score matching depending on the primary tumor type. NSCLC, non-small cell lung cancer, SCLC, small cell lung cancer, PTS, primary tumor surgery.


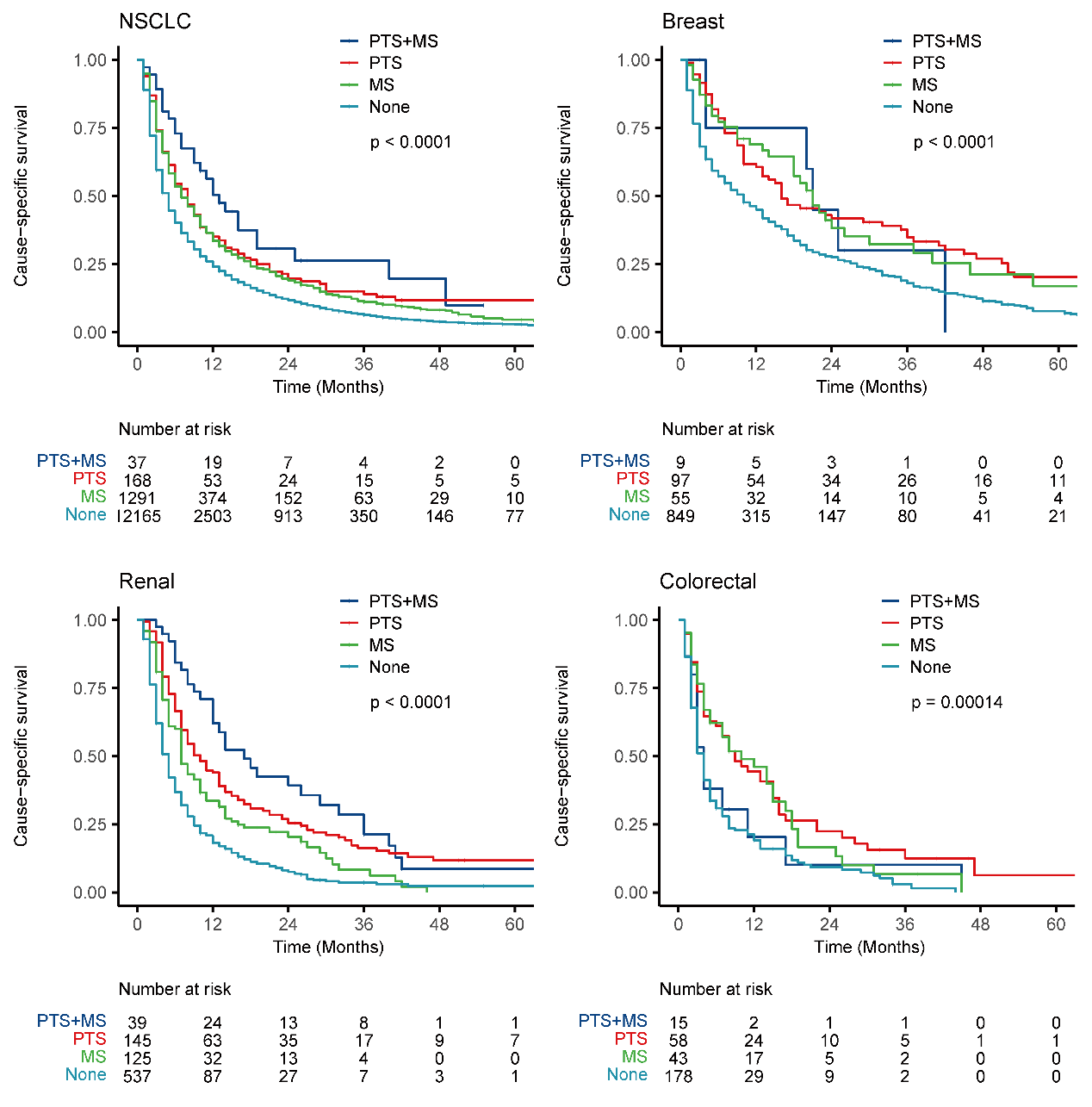


Figure S3: Kaplan-Meier curves for cause-specific survival of patients with intracranial-extracranial metastases by surgical sites after propensity score matching depending on the primary tumor type. PTS primary tumor surgery, MS metastatic surgery, NSCLC non-small cell lung cancer.

Table S1: Baseline characteristics of small cell lung cancer patients with brain metastases grouped by primary tumor surgery before and after propensity score matching.

|  | **Before matching** | | | **After matching** | | |
| --- | --- | --- | --- | --- | --- | --- |
|  | **No PTS** | **PTS** | ***p* value** | **No PTS** | **PTS** | ***p* value** |
|  | **N=5296** | **N=49** |  | **N=46** | **N=46** |  |
| Age: |  |  | 0.213 |  |  | 0.899 |
| <60 | 1523 (28.8%) | 16 (32.7%) |  | 13 (28.3%) | 15 (32.6%) |  |
| 60-69 | 2072 (39.1%) | 23 (46.9%) |  | 22 (47.8%) | 21 (45.7%) |  |
| >70 | 1701 (32.1%) | 10 (20.4%) |  | 11 (23.9%) | 10 (21.7%) |  |
| Sex: |  |  | 0.461 |  |  | 0.525 |
| Female | 2476 (46.8%) | 26 (53.1%) |  | 29 (63.0%) | 25 (54.3%) |  |
| Male | 2820 (53.2%) | 23 (46.9%) |  | 17 (37.0%) | 21 (45.7%) |  |
| Race: |  |  | 0.123 |  |  | 0.259 |
| Black | 607 (11.5%) | 10 (20.4%) |  | 5 (10.9%) | 9 (19.6%) |  |
| White | 4447 (84.0%) | 38 (77.6%) |  | 41 (89.1%) | 36 (78.3%) |  |
| Other | 242 (4.57%) | 1 (2.04%) |  | 0 (0.00%) | 1 (2.17%) |  |
| Year of diagnosis: |  |  | 0.253 |  |  | 0.961 |
| 2010-2012 | 1667 (31.5%) | 14 (28.6%) |  | 11 (23.9%) | 12 (26.1%) |  |
| 2013-2015 | 1798 (34.0%) | 22 (44.9%) |  | 21 (45.7%) | 21 (45.7%) |  |
| 2016-2018 | 1831 (34.6%) | 13 (26.5%) |  | 14 (30.4%) | 13 (28.3%) |  |
| T stage: |  |  | 0.043 |  |  | 0.497 |
| T1-T2 | 1657 (31.3%) | 23 (46.9%) |  | 23 (50.0%) | 20 (43.5%) |  |
| T3-T4 | 2936 (55.4%) | 23 (46.9%) |  | 18 (39.1%) | 23 (50.0%) |  |
| Tx | 703 (13.3%) | 3 (6.12%) |  | 5 (10.9%) | 3 (6.52%) |  |
| N stage: |  |  | 0.843 |  |  | 0.849 |
| N0 | 727 (13.7%) | 8 (16.3%) |  | 11 (23.9%) | 8 (17.4%) |  |
| N1-N3 | 4292 (81.0%) | 39 (79.6%) |  | 33 (71.7%) | 36 (78.3%) |  |
| Nx | 277 (5.23%) | 2 (4.08%) |  | 2 (4.35%) | 2 (4.35%) |  |
| Grade: |  |  | 0.192 |  |  | 1.000 |
| 1 | 8 (0.15%) | 0 (0.00%) |  | 0 (0%) | 0 (0%) |  |
| 2 | 15 (0.28%) | 0 (0.00%) |  | 0 (0%) | 0 (0%) |  |
| 3 | 408 (7.70%) | 6 (12.2%) |  | 5 (10.9%) | 5 (10.9%) |  |
| 4 | 560 (10.6%) | 9 (18.4%) |  | 7 (15.2%) | 7 (15.2%) |  |
| Unknown | 4305 (81.3%) | 34 (69.4%) |  | 34 (73.9%) | 34 (73.9%) |  |
| Lung metastasis: |  |  | 0.067 |  |  | 1.000 |
| No | 4395 (83.0%) | 46 (93.9%) |  | 44 (95.7%) | 43 (93.5%) |  |
| Yes | 901 (17.0%) | 3 (6.12%) |  | 2 (4.35%) | 3 (6.52%) |  |
| Liver metastasis: |  |  | 0.003 |  |  | 1.000 |
| No | 3548 (67.0%) | 43 (87.8%) |  | 41 (89.1%) | 40 (87.0%) |  |
| Yes | 1748 (33.0%) | 6 (12.2%) |  | 5 (10.9%) | 6 (13.0%) |  |
| Bone metastasis: |  |  | 0.001 |  |  | 1.000 |
| No | 3727 (70.4%) | 46 (93.9%) |  | 44 (95.7%) | 43 (93.5%) |  |
| Yes | 1569 (29.6%) | 3 (6.12%) |  | 2 (4.35%) | 3 (6.52%) |  |
| Chemotherapy: |  |  | 0.566 |  |  | 0.807 |
| No | 1550 (29.3%) | 12 (24.5%) |  | 10 (21.7%) | 12 (26.1%) |  |
| Yes | 3746 (70.7%) | 37 (75.5%) |  | 36 (78.3%) | 34 (73.9%) |  |
| Radiation: |  |  | 0.391 |  |  | 0.800 |
| No | 1539 (29.1%) | 11 (22.4%) |  | 9 (19.6%) | 11 (23.9%) |  |
| Yes | 3757 (70.9%) | 38 (77.6%) |  | 37 (80.4%) | 35 (76.1%) |  |
| Metastatic surgery: |  |  | 0.015 |  |  | 0.738 |
| No | 4945 (93.4%) | 41 (83.7%) |  | 42 (91.3%) | 40 (87.0%) |  |
| Yes | 351 (6.63%) | 8 (16.3%) |  | 4 (8.70%) | 6 (13.0%) |  |

PTS, primary tumor surgery.

Table S2: Baseline characteristics of breast cancer patients with brain metastases grouped by primary tumor surgery before and after propensity score matching.

|  | **Before matching** | | | **After matching** | | |
| --- | --- | --- | --- | --- | --- | --- |
|  | **No PTS** | **PTS** | ***p* value** | **No PTS** | **PTS** | ***p* value** |
|  | **N=1047** | **N=160** |  | **N=160** | **N=160** |  |
| Age: |  |  | 0.097 |  |  | 0.627 |
| <60 | 479 (45.7%) | 87 (54.4%) |  | 84 (52.5%) | 87 (54.4%) |  |
| 60-69 | 338 (32.3%) | 40 (25.0%) |  | 36 (22.5%) | 40 (25.0%) |  |
| >70 | 230 (22.0%) | 33 (20.6%) |  | 40 (25.0%) | 33 (20.6%) |  |
| Sex: |  |  | 0.253 |  |  | 1.000 |
| Female | 1035 (98.9%) | 156 (97.5%) |  | 157 (98.1%) | 156 (97.5%) |  |
| Male | 12 (1.15%) | 4 (2.50%) |  | 3 (1.88%) | 4 (2.50%) |  |
| Race: |  |  | 0.860 |  |  | 0.938 |
| Black | 229 (21.9%) | 37 (23.1%) |  | 39 (24.4%) | 37 (23.1%) |  |
| White | 735 (70.2%) | 109 (68.1%) |  | 106 (66.2%) | 109 (68.1%) |  |
| Other | 83 (7.93%) | 14 (8.75%) |  | 15 (9.38%) | 14 (8.75%) |  |
| Year of diagnosis: |  |  | <0.001 |  |  | 0.784 |
| 2010-2012 | 304 (29.0%) | 76 (47.5%) |  | 75 (46.9%) | 76 (47.5%) |  |
| 2013-2015 | 366 (35.0%) | 47 (29.4%) |  | 52 (32.5%) | 47 (29.4%) |  |
| 2016-2018 | 377 (36.0%) | 37 (23.1%) |  | 33 (20.6%) | 37 (23.1%) |  |
| Subtype: |  |  | 0.002 |  |  | 0.665 |
| HR-/HER2- | 181 (17.3%) | 47 (29.4%) |  | 39 (24.4%) | 47 (29.4%) |  |
| HR-/HER2+ | 129 (12.3%) | 18 (11.2%) |  | 17 (10.6%) | 18 (11.2%) |  |
| HR+/HER2- | 410 (39.2%) | 61 (38.1%) |  | 70 (43.8%) | 61 (38.1%) |  |
| HR+/HER2+ | 179 (17.1%) | 23 (14.4%) |  | 19 (11.9%) | 23 (14.4%) |  |
| Other breast cancer | 148 (14.1%) | 11 (6.88%) |  | 15 (9.38%) | 11 (6.88%) |  |
| T stage: |  |  | <0.001 |  |  | 0.822 |
| T1-T2 | 317 (30.3%) | 70 (43.8%) |  | 66 (41.2%) | 70 (43.8%) |  |
| T3-T4 | 525 (50.1%) | 83 (51.9%) |  | 85 (53.1%) | 83 (51.9%) |  |
| Tx | 205 (19.6%) | 7 (4.38%) |  | 9 (5.62%) | 7 (4.38%) |  |
| N stage: |  |  | 0.016 |  |  | 0.963 |
| N0 | 230 (22.0%) | 31 (19.4%) |  | 32 (20.0%) | 31 (19.4%) |  |
| N1-N3 | 684 (65.3%) | 120 (75.0%) |  | 120 (75.0%) | 120 (75.0%) |  |
| Nx | 133 (12.7%) | 9 (5.62%) |  | 8 (5.00%) | 9 (5.62%) |  |
| Grade: |  |  | <0.001 |  |  | 0.919 |
| 1 | 36 (3.44%) | 7 (4.38%) |  | 10 (6.25%) | 7 (4.38%) |  |
| 2 | 251 (24.0%) | 32 (20.0%) |  | 28 (17.5%) | 32 (20.0%) |  |
| 3 | 368 (35.1%) | 100 (62.5%) |  | 99 (61.9%) | 100 (62.5%) |  |
| 4 | 10 (0.96%) | 1 (0.62%) |  | 1 (0.62%) | 1 (0.62%) |  |
| Unknown | 382 (36.5%) | 20 (12.5%) |  | 22 (13.8%) | 20 (12.5%) |  |
| Lung metastasis: |  |  | 0.018 |  |  | 1.000 |
| No | 552 (52.7%) | 101 (63.1%) |  | 100 (62.5%) | 101 (63.1%) |  |
| Yes | 495 (47.3%) | 59 (36.9%) |  | 60 (37.5%) | 59 (36.9%) |  |
| Liver metastasis: |  |  | <0.001 |  |  | 1.000 |
| No | 661 (63.1%) | 130 (81.2%) |  | 129 (80.6%) | 130 (81.2%) |  |
| Yes | 386 (36.9%) | 30 (18.8%) |  | 31 (19.4%) | 30 (18.8%) |  |
| Bone metastasis: |  |  | <0.001 |  |  | 0.911 |
| No | 330 (31.5%) | 81 (50.6%) |  | 79 (49.4%) | 81 (50.6%) |  |
| Yes | 717 (68.5%) | 79 (49.4%) |  | 81 (50.6%) | 79 (49.4%) |  |
| Chemotherapy: |  |  | <0.001 |  |  | 1.000 |
| No | 496 (47.4%) | 46 (28.7%) |  | 47 (29.4%) | 46 (28.7%) |  |
| Yes | 551 (52.6%) | 114 (71.2%) |  | 113 (70.6%) | 114 (71.2%) |  |
| Radiation: |  |  | 0.001 |  |  | 0.362 |
| No | 376 (35.9%) | 35 (21.9%) |  | 43 (26.9%) | 35 (21.9%) |  |
| Yes | 671 (64.1%) | 125 (78.1%) |  | 117 (73.1%) | 125 (78.1%) |  |
| Metastatic surgery |  |  | 0.017 |  |  | 0.216 |
| No | 966 (92.3%) | 138 (86.2%) |  | 146 (91.2%) | 138 (86.2%) |  |
| Yes | 81 (7.74%) | 22 (13.8%) |  | 14 (8.75%) | 22 (13.8%) |  |

PTS primary tumor surgery, HR hormone receptor, HER2 human epidermal growth factor receptor 2.

Table S3: Baseline characteristics of renal cancer patients with brain metastases grouped by primary tumor surgery before and after propensity score matching.

|  | **Before matching** | | | **After matching** | | |
| --- | --- | --- | --- | --- | --- | --- |
|  | **No PTS** | **PTS** | ***p* value** | **No PTS** | **PTS** | ***p* value** |
|  | **N=744** | **N=266** |  | **N=178** | **N=178** |  |
| Age: |  |  | <0.001 |  |  | 0.551 |
| <60 | 306 (41.1%) | 138 (51.9%) |  | 80 (44.9%) | 88 (49.4%) |  |
| 60-69 | 270 (36.3%) | 96 (36.1%) |  | 64 (36.0%) | 63 (35.4%) |  |
| >70 | 168 (22.6%) | 32 (12.0%) |  | 34 (19.1%) | 27 (15.2%) |  |
| Sex: |  |  | 0.847 |  |  | 0.724 |
| Female | 236 (31.7%) | 82 (30.8%) |  | 48 (27.0%) | 52 (29.2%) |  |
| Male | 508 (68.3%) | 184 (69.2%) |  | 130 (73.0%) | 126 (70.8%) |  |
| Race: |  |  | 0.071 |  |  | 0.796 |
| Black | 73 (9.81%) | 14 (5.26%) |  | 9 (5.06%) | 12 (6.74%) |  |
| White | 620 (83.3%) | 231 (86.8%) |  | 157 (88.2%) | 154 (86.5%) |  |
| Other | 51 (6.85%) | 21 (7.89%) |  | 12 (6.74%) | 12 (6.74%) |  |
| Year of diagnosis: |  |  | 0.001 |  |  | 0.346 |
| 2010-2012 | 243 (32.7%) | 83 (31.2%) |  | 47 (26.4%) | 58 (32.6%) |  |
| 2013-2015 | 221 (29.7%) | 110 (41.4%) |  | 74 (41.6%) | 73 (41.0%) |  |
| 2016-2018 | 280 (37.6%) | 73 (27.4%) |  | 57 (32.0%) | 47 (26.4%) |  |
| subtype: |  |  | <0.001 |  |  | 0.862 |
| Clear cell adenocarcinoma | 318 (42.7%) | 180 (67.7%) |  | 106 (59.6%) | 111 (62.4%) |  |
| Other | 82 (11.0%) | 31 (11.7%) |  | 25 (14.0%) | 23 (12.9%) |  |
| Renal cell carcinoma | 344 (46.2%) | 55 (20.7%) |  | 47 (26.4%) | 44 (24.7%) |  |
| T stage: |  |  | <0.001 |  |  | 0.355 |
| T1-T2 | 338 (45.4%) | 73 (27.4%) |  | 70 (39.3%) | 62 (34.8%) |  |
| T3-T4 | 232 (31.2%) | 186 (69.9%) |  | 97 (54.5%) | 109 (61.2%) |  |
| Tx | 174 (23.4%) | 7 (2.63%) |  | 11 (6.18%) | 7 (3.93%) |  |
| N stage: |  |  | <0.001 |  |  | 0.116 |
| N0 | 405 (54.4%) | 183 (68.8%) |  | 106 (59.6%) | 123 (69.1%) |  |
| N1-N3 | 218 (29.3%) | 62 (23.3%) |  | 56 (31.5%) | 39 (21.9%) |  |
| Nx | 121 (16.3%) | 21 (7.89%) |  | 16 (8.99%) | 16 (8.99%) |  |
| Grade: |  |  | . |  |  | 0.079 |
| 1 | 11 (1.48%) | 2 (0.75%) |  | 1 (0.56%) | 2 (1.12%) |  |
| 2 | 52 (6.99%) | 30 (11.3%) |  | 34 (19.1%) | 25 (14.0%) |  |
| 3 | 62 (8.33%) | 93 (35.0%) |  | 50 (28.1%) | 59 (33.1%) |  |
| 4 | 30 (4.03%) | 90 (33.8%) |  | 27 (15.2%) | 42 (23.6%) |  |
| Unknown | 589 (79.2%) | 51 (19.2%) |  | 66 (37.1%) | 50 (28.1%) |  |
| Lung metastasis: |  |  | <0.001 |  |  | 0.124 |
| No | 179 (24.1%) | 115 (43.2%) |  | 58 (32.6%) | 73 (41.0%) |  |
| Yes | 565 (75.9%) | 151 (56.8%) |  | 120 (67.4%) | 105 (59.0%) |  |
| Liver metastasis: |  |  | <0.001 |  |  | 0.364 |
| No | 567 (76.2%) | 238 (89.5%) |  | 149 (83.7%) | 156 (87.6%) |  |
| Yes | 177 (23.8%) | 28 (10.5%) |  | 29 (16.3%) | 22 (12.4%) |  |
| Bone metastasis: |  |  | <0.001 |  |  | 1.000 |
| No | 416 (55.9%) | 188 (70.7%) |  | 121 (68.0%) | 122 (68.5%) |  |
| Yes | 328 (44.1%) | 78 (29.3%) |  | 57 (32.0%) | 56 (31.5%) |  |
| Chemotherapy: |  |  | 0.670 |  |  | 0.595 |
| No | 356 (47.8%) | 132 (49.6%) |  | 80 (44.9%) | 86 (48.3%) |  |
| Yes | 388 (52.2%) | 134 (50.4%) |  | 98 (55.1%) | 92 (51.7%) |  |
| Radiation: |  |  | 0.095 |  |  | 1.000 |
| No | 191 (25.7%) | 54 (20.3%) |  | 42 (23.6%) | 41 (23.0%) |  |
| Yes | 553 (74.3%) | 212 (79.7%) |  | 136 (76.4%) | 137 (77.0%) |  |
| Metastatic surgery: |  |  | 0.014 |  |  | 0.714 |
| No | 593 (79.7%) | 192 (72.2%) |  | 135 (75.8%) | 131 (73.6%) |  |
| Yes | 151 (20.3%) | 74 (27.8%) |  | 43 (24.2%) | 47 (26.4%) |  |

PTS primary tumor surgery.

Table S4: Baseline characteristics of melanoma patients with brain metastases grouped by primary tumor surgery before and after propensity score matching.

|  | **Before matching** | | | **After matching** | | |
| --- | --- | --- | --- | --- | --- | --- |
|  | **No PTS** | **PTS** | ***p* value** | **No PTS** | **PTS** | ***p* value** |
|  | **N= 416** | **N= 218** |  | **N= 90** | **N= 90** |  |
| Age: |  |  | 0.180 |  |  | 0.915 |
| <60 | 178 (42.8%) | 105 (48.2%) |  | 42 (46.7%) | 42 (46.7%) |  |
| 60-69 | 106 (25.5%) | 59 (27.1%) |  | 31 (34.4%) | 29 (32.2%) |  |
| >70 | 132 (31.7%) | 54 (24.8%) |  | 17 (18.9%) | 19 (21.1%) |  |
| Sex: |  |  | 1.000 |  |  | 0.879 |
| Female | 123 (29.6%) | 65 (29.8%) |  | 37 (41.1%) | 35 (38.9%) |  |
| Male | 293 (70.4%) | 153 (70.2%) |  | 53 (58.9%) | 55 (61.1%) |  |
| Race: |  |  | 0.104 |  |  | 1.000 |
| Black | 3 (0.72%) | 1 (0.46%) |  | 0 (0.00%) | 1 (1.11%) |  |
| White | 398 (95.7%) | 215 (98.6%) |  | 88 (97.8%) | 87 (96.7%) |  |
| Other | 15 (3.61%) | 2 (0.92%) |  | 2 (2.22%) | 2 (2.22%) |  |
| Year of diagnosis: |  |  | 0.031 |  |  | 0.625 |
| 2010-2012 | 96 (23.1%) | 62 (28.4%) |  | 25 (27.8%) | 31 (34.4%) |  |
| 2013-2015 | 126 (30.3%) | 78 (35.8%) |  | 28 (31.1%) | 25 (27.8%) |  |
| 2016-2018 | 194 (46.6%) | 78 (35.8%) |  | 37 (41.1%) | 34 (37.8%) |  |
| T stage: |  |  | <0.001 |  |  | 0.466 |
| T1-T2 | 18 (4.33%) | 41 (18.8%) |  | 15 (16.7%) | 12 (13.3%) |  |
| T3-T4 | 20 (4.81%) | 126 (57.8%) |  | 20 (22.2%) | 27 (30.0%) |  |
| Tx | 378 (90.9%) | 51 (23.4%) |  | 55 (61.1%) | 51 (56.7%) |  |
| N stage: |  |  | <0.001 |  |  | 0.748 |
| N0 | 121 (29.1%) | 97 (44.5%) |  | 40 (44.4%) | 42 (46.7%) |  |
| N1-N3 | 85 (20.4%) | 101 (46.3%) |  | 31 (34.4%) | 33 (36.7%) |  |
| Nx | 210 (50.5%) | 20 (9.17%) |  | 19 (21.1%) | 15 (16.7%) |  |
| Grade: |  |  | 0.783 |  |  | 1.000 |
| 1 | 1 (0.24%) | 0 (0.00%) |  | 0 (0.00%) | 0 (0.00%) |  |
| 3 | 5 (1.20%) | 1 (0.46%) |  | 0 (0.00%) | 1 (1.11%) |  |
| Unknown | 410 (98.6%) | 217 (99.5%) |  | 90 (100%) | 89 (98.9%) |  |
| Lung metastasis: |  |  | 0.001 |  |  | 1.000 |
| No | 175 (42.1%) | 124 (56.9%) |  | 46 (51.1%) | 47 (52.2%) |  |
| Yes | 241 (57.9%) | 94 (43.1%) |  | 44 (48.9%) | 43 (47.8%) |  |
| Liver metastasis: |  |  | 0.500 |  |  | 0.613 |
| No | 309 (74.3%) | 168 (77.1%) |  | 64 (71.1%) | 68 (75.6%) |  |
| Yes | 107 (25.7%) | 50 (22.9%) |  | 26 (28.9%) | 22 (24.4%) |  |
| Bone metastasis: |  |  | 0.050 |  |  | 0.491 |
| No | 299 (71.9%) | 173 (79.4%) |  | 65 (72.2%) | 70 (77.8%) |  |
| Yes | 117 (28.1%) | 45 (20.6%) |  | 25 (27.8%) | 20 (22.2%) |  |
| Chemotherapy: |  |  | 0.606 |  |  | 0.626 |
| No | 322 (77.4%) | 164 (75.2%) |  | 65 (72.2%) | 61 (67.8%) |  |
| Yes | 94 (22.6%) | 54 (24.8%) |  | 25 (27.8%) | 29 (32.2%) |  |
| Radiation: |  |  | 0.607 |  |  | 0.535 |
| No | 122 (29.3%) | 69 (31.7%) |  | 30 (33.3%) | 35 (38.9%) |  |
| Yes | 294 (70.7%) | 149 (68.3%) |  | 60 (66.7%) | 55 (61.1%) |  |
| Metastatic surgery |  |  | 0.001 |  |  | 0.236 |
| No | 298 (71.6%) | 184 (84.4%) |  | 71 (78.9%) | 78 (86.7%) |  |
| Yes | 118 (28.4%) | 34 (15.6%) |  | 19 (21.1%) | 12 (13.3%) |  |

PTS primary tumor surgery.

Table S5: Baseline characteristics of colorectal cancer patients with brain metastases grouped by primary tumor surgery before and after propensity score matching.

|  | **Before matching** | | | **After matching** | | |
| --- | --- | --- | --- | --- | --- | --- |
|  | **No PTS** | **PTS** | ***p* value** | **No PTS** | **PTS** | ***p* value** |
|  | **N=249** | **N=130** |  | **N= 45** | **N= 45** |  |
| Age: |  |  | 0.645 |  |  | 0.909 |
| <60 | 106 (42.6%) | 49 (37.7%) |  | 16 (35.6%) | 18 (40.0%) |  |
| 60-69 | 67 (26.9%) | 39 (30.0%) |  | 17 (37.8%) | 16 (35.6%) |  |
| >70 | 76 (30.5%) | 42 (32.3%) |  | 12 (26.7%) | 11 (24.4%) |  |
| Sex: |  |  | 0.879 |  |  | 1.000 |
| Female | 121 (48.6%) | 65 (50.0%) |  | 21 (46.7%) | 20 (44.4%) |  |
| Male | 128 (51.4%) | 65 (50.0%) |  | 24 (53.3%) | 25 (55.6%) |  |
| Race: |  |  | 0.969 |  |  | 1.000 |
| Black | 40 (16.1%) | 21 (16.2%) |  | 8 (17.8%) | 9 (20.0%) |  |
| White | 190 (76.3%) | 100 (76.9%) |  | 34 (75.6%) | 33 (73.3%) |  |
| Other | 19 (7.63%) | 9 (6.92%) |  | 3 (6.67%) | 3 (6.67%) |  |
| Year of diagnosis: |  |  | 0.014 |  |  | 0.467 |
| 2010-2012 | 80 (32.1%) | 56 (43.1%) |  | 18 (40.0%) | 22 (48.9%) |  |
| 2013-2015 | 69 (27.7%) | 41 (31.5%) |  | 13 (28.9%) | 14 (31.1%) |  |
| 2016-2018 | 100 (40.2%) | 33 (25.4%) |  | 14 (31.1%) | 9 (20.0%) |  |
| T stage: |  |  | <0.001 |  |  | 0.147 |
| T1-T2 | 39 (15.7%) | 7 (5.38%) |  | 11 (24.4%) | 7 (15.6%) |  |
| T3-T4 | 34 (13.7%) | 117 (90.0%) |  | 23 (51.1%) | 32 (71.1%) |  |
| Tx | 176 (70.7%) | 6 (4.62%) |  | 11 (24.4%) | 6 (13.3%) |  |
| N stage: |  |  | <0.001 |  |  | 0.477 |
| N0 | 89 (35.7%) | 32 (24.6%) |  | 18 (40.0%) | 17 (37.8%) |  |
| N1-N3 | 70 (28.1%) | 96 (73.8%) |  | 22 (48.9%) | 26 (57.8%) |  |
| Nx | 90 (36.1%) | 2 (1.54%) |  | 5 (11.1%) | 2 (4.44%) |  |
| Grade: |  |  | <0.001 |  |  | 0.258 |
| 1 | 3 (1.20%) | 4 (3.08%) |  | 2 (4.44%) | 3 (6.67%) |  |
| 2 | 66 (26.5%) | 68 (52.3%) |  | 18 (40.0%) | 18 (40.0%) |  |
| 3 | 36 (14.5%) | 37 (28.5%) |  | 10 (22.2%) | 10 (22.2%) |  |
| 4 | 2 (0.80%) | 10 (7.69%) |  | 1 (2.22%) | 6 (13.3%) |  |
| Unknown | 142 (57.0%) | 11 (8.46%) |  | 14 (31.1%) | 8 (17.8%) |  |
| Lung metastasis: |  |  | <0.001 |  |  | 0.826 |
| No | 99 (39.8%) | 91 (70.0%) |  | 28 (62.2%) | 30 (66.7%) |  |
| Yes | 150 (60.2%) | 39 (30.0%) |  | 17 (37.8%) | 15 (33.3%) |  |
| Liver metastasis: |  |  | <0.001 |  |  | 0.830 |
| No | 77 (30.9%) | 74 (56.9%) |  | 17 (37.8%) | 19 (42.2%) |  |
| Yes | 172 (69.1%) | 56 (43.1%) |  | 28 (62.2%) | 26 (57.8%) |  |
| Bone metastasis: |  |  | <0.001 |  |  | 1.000 |
| No | 172 (69.1%) | 120 (92.3%) |  | 35 (77.8%) | 36 (80.0%) |  |
| Yes | 77 (30.9%) | 10 (7.69%) |  | 10 (22.2%) | 9 (20.0%) |  |
| Chemotherapy: |  |  | 0.619 |  |  | 0.672 |
| No | 125 (50.2%) | 61 (46.9%) |  | 26 (57.8%) | 23 (51.1%) |  |
| Yes | 124 (49.8%) | 69 (53.1%) |  | 19 (42.2%) | 22 (48.9%) |  |
| Radiation: |  |  | 0.218 |  |  | 0.833 |
| No | 99 (39.8%) | 61 (46.9%) |  | 20 (44.4%) | 22 (48.9%) |  |
| Yes | 150 (60.2%) | 69 (53.1%) |  | 25 (55.6%) | 23 (51.1%) |  |
| Metastatic surgery: |  |  | 0.102 |  |  | 1.000 |
| No | 193 (77.5%) | 90 (69.2%) |  | 36 (80.0%) | 37 (82.2%) |  |
| Yes | 56 (22.5%) | 40 (30.8%) |  | 9 (20.0%) | 8 (17.8%) |  |

PTS, primary tumor surgery.

Table S6: Baseline characteristics of liver cancer patients with brain metastases grouped by primary tumor surgery before and after propensity score matching.

|  | **Before matching** | | | **After matching** | | |
| --- | --- | --- | --- | --- | --- | --- |
|  | **No PTS** | **PTS** | ***p* value** | **No PTS** | **PTS** | ***p* value** |
|  | **N=141** | **N=12** |  | **N=12** | **N=12** |  |
| Age: |  |  | 1.000 |  |  | 1.000 |
| <60 | 49 (34.8%) | 4 (33.3%) |  | 5 (41.7%) | 4 (33.3%) |  |
| 60-69 | 51 (36.2%) | 5 (41.7%) |  | 5 (41.7%) | 5 (41.7%) |  |
| >70 | 41 (29.1%) | 3 (25.0%) |  | 2 (16.7%) | 3 (25.0%) |  |
| Sex: |  |  | 0.097 |  |  | 0.400 |
| Female | 37 (26.2%) | 6 (50.0%) |  | 3 (25.0%) | 6 (50.0%) |  |
| Male | 104 (73.8%) | 6 (50.0%) |  | 9 (75.0%) | 6 (50.0%) |  |
| Race: |  |  | 0.498 |  |  | 1.000 |
| Black | 21 (14.9%) | 0 (0.00%) |  | 0 (0.00%) | 0 (0.00%) |  |
| White | 100 (70.9%) | 10 (83.3%) |  | 10 (83.3%) | 10 (83.3%) |  |
| Other | 20 (14.2%) | 2 (16.7%) |  | 2 (16.7%) | 2 (16.7%) |  |
| Year of diagnosis: |  |  | 0.156 |  |  | 1.000 |
| 2010-2012 | 33 (23.4%) | 6 (50.0%) |  | 6 (50.0%) | 6 (50.0%) |  |
| 2013-2015 | 52 (36.9%) | 3 (25.0%) |  | 2 (16.7%) | 3 (25.0%) |  |
| 2016-2018 | 56 (39.7%) | 3 (25.0%) |  | 4 (33.3%) | 3 (25.0%) |  |
| Subtype: |  |  | 0.014 |  |  | 0.400 |
| Cholangiocarcinoma | 29 (20.6%) | 0 (0.00%) |  | 0 (0.00%) | 0 (0.00%) |  |
| Hepatocellular carcinoma | 89 (63s.1%) | 6 (50.0%) |  | 9 (75.0%) | 6 (50.0%) |  |
| Other | 23 (16.3%) | 6 (50.0%) |  | 3 (25.0%) | 6 (50.0%) |  |
| T stage: |  |  | 0.484 |  |  | 0.589 |
| T1-T2 | 41 (29.1%) | 3 (25.0%) |  | 4 (33.3%) | 3 (25.0%) |  |
| T3-T4 | 47 (33.3%) | 6 (50.0%) |  | 3 (25.0%) | 6 (50.0%) |  |
| Tx | 53 (37.6%) | 3 (25.0%) |  | 5 (41.7%) | 3 (25.0%) |  |
| N stage: |  |  | 0.646 |  |  | 1.000 |
| N0 | 82 (58.2%) | 9 (75.0%) |  | 10 (83.3%) | 9 (75.0%) |  |
| N1-N3 | 30 (21.3%) | 2 (16.7%) |  | 1 (8.33%) | 2 (16.7%) |  |
| Nx | 29 (20.6%) | 1 (8.33%) |  | 1 (8.33%) | 1 (8.33%) |  |
| Grade: |  |  | 0.017 |  |  | 1.000 |
| 1 | 11 (7.80%) | 0 (0.00%) |  | 0 (0.00%) | 0 (0.00%) |  |
| 2 | 23 (16.3%) | 2 (16.7%) |  | 3 (25.0%) | 2 (16.7%) |  |
| 3 | 15 (10.6%) | 5 (41.7%) |  | 4 (33.3%) | 5 (41.7%) |  |
| 4 | 3 (2.13%) | 1 (8.33%) |  | 1 (8.33%) | 1 (8.33%) |  |
| Unknown | 89 (63.1%) | 4 (33.3%) |  | 4 (33.3%) | 4 (33.3%) |  |
| Lung metastasis: |  |  | 0.537 |  |  | 1.000 |
| No | 86 (61.0%) | 9 (75.0%) |  | 9 (75.0%) | 9 (75.0%) |  |
| Yes | 55 (39.0%) | 3 (25.0%) |  | 3 (25.0%) | 3 (25.0%) |  |
| Liver metastasis: |  |  | 0.485 |  |  | 0.640 |
| No | 108 (76.6%) | 8 (66.7%) |  | 10 (83.3%) | 8 (66.7%) |  |
| Yes | 33 (23.4%) | 4 (33.3%) |  | 2 (16.7%) | 4 (33.3%) |  |
| Bone metastasis: |  |  | 0.553 |  |  | 0.679 |
| No | 84 (59.6%) | 6 (50.0%) |  | 8 (66.7%) | 6 (50.0%) |  |
| Yes | 57 (40.4%) | 6 (50.0%) |  | 4 (33.3%) | 6 (50.0%) |  |
| Chemotherapy: |  |  | 0.537 |  |  | 1.000 |
| No | 86 (61.0%) | 9 (75.0%) |  | 9 (75.0%) | 9 (75.0%) |  |
| Yes | 55 (39.0%) | 3 (25.0%) |  | 3 (25.0%) | 3 (25.0%) |  |
| Radiation: |  |  | 1.000 |  |  | 1.000 |
| No | 75 (53.2%) | 6 (50.0%) |  | 6 (50.0%) | 6 (50.0%) |  |
| Yes | 66 (46.8%) | 6 (50.0%) |  | 6 (50.0%) | 6 (50.0%) |  |
| Metastatic surgery: |  |  | 1.000 |  |  | 0.640 |
| No | 117 (83.0%) | 10 (83.3%) |  | 8 (66.7%) | 10 (83.3%) |  |
| Yes | 24 (17.0%) | 2 (16.7%) |  | 4 (33.3%) | 2 (16.7%) |  |

PTS, primary tumor surgery.
